# PDP-CON: prediction of domain/linker residues in protein sequences using a consensus approach

**DOI:** 10.1007/s00894-016-2933-0

**Published:** 2016-03-11

**Authors:** Piyali Chatterjee, Subhadip Basu, Julian Zubek, Mahantapas Kundu, Mita Nasipuri, Dariusz Plewczynski

**Affiliations:** Department of Computer Science and Engineering, Netaji Subhash Engineering College, Garia, Kolkata, 700152 India; Department of Computer Science and Engineering, Jadavpur University, Kolkata, 700032 India; Institute of Computer Science, Polish Academy of Sciences, Warsaw, Poland; Center of New Technologies, University of Warsaw, Banacha 2c, 02-097 Warsaw, Poland; Faculty of Pharmacy, Medical University of Warsaw, Warsaw, Poland

**Keywords:** Protein domain/linker prediction, Domain boundary prediction, Physicochemical properties, Ordered-disordered regions in protein sequence, Machine-learning approaches, Consensus strategy

## Abstract

The prediction of domain/linker residues in protein sequences is a crucial task in the functional classification of proteins, homology-based protein structure prediction, and high-throughput structural genomics. In this work, a novel consensus-based machine-learning technique was applied for residue-level prediction of the domain/linker annotations in protein sequences using ordered/disordered regions along protein chains and a set of physicochemical properties. Six different classifiers—decision tree, Gaussian naïve Bayes, linear discriminant analysis, support vector machine, random forest, and multilayer perceptron—were exhaustively explored for the residue-level prediction of domain/linker regions. The protein sequences from the curated CATH database were used for training and cross-validation experiments. Test results obtained by applying the developed PDP-CON tool to the mutually exclusive, independent proteins of the CASP-8, CASP-9, and CASP-10 databases are reported. An *n*-star quality consensus approach was used to combine the results yielded by different classifiers. The average PDP-CON accuracy and F-measure values for the CASP targets were found to be 0.86 and 0.91, respectively. The dataset, source code, and all supplementary materials for this work are available at https://cmaterju.org/cmaterbioinfo/ for noncommercial use.

**Electronic supplementary material:**

The online version of this article (doi:10.1007/s00894-016-2933-0) contains supplementary material, which is available to authorized users.

## Introduction

Some simple combinations of protein secondary-structural elements that are found to occur frequently in proteins are referred to as *super-secondary structures* or *motifs*. Several motifs pack together to form compact, local, semi-independent units called *domains*. A domain is a segment of a polypeptide chain that can fold into a three-dimensional structure irrespective of the presence of other segments of the chain [1]. The overall 3D structure of a protein’s polypeptide chain is referred to as its tertiary structure, whereas the domain is the fundamental building block of tertiary structure. Each domain contains a hydrophobic core built from secondary-structural units connected by loop regions. Two-thirds of the proteins in unicellular organisms and more than 80 % of those in metazoans are multidomain proteins created as a result of gene duplication events. As the complexity of an organism increases, the number of domains in its proteins increases. Multidomain proteins are likely to have emerged during evolution as a consequence of selective pressure to create new functions. Various proteins have diverged from common ancestors by presenting different combinations and associations of domains. To predict the tertiary structure of a protein, it is useful to segment the protein by identifying the domain boundaries in it. The resulting knowledge of the domains of the protein can be used to classify it and understand its structure, function, and evolution. A number of methods have been developed to identify multidomains in protein chains from their primary sequences, are discussed below.

Galzitskaya et al. [2] developed a method based on the idea that the 3D structure of the protein is a result of a balance between maximizing the attractive native interactions present and minimizing the loss of conformational entropy (i.e., the topology of the chain determines how much chain entropy is lost as the native interactions are formed). Thus, domain boundary prediction involved finding the minima in a latent entropy profile. When regions with high entropy are unfolded, they form many residue–residue interactions to compensate for the loss of entropy. Such regions are independent folding units; i.e., domains. Those authors considered the conformational entropy of each amino acid and searched for the global minimum in the entropy profile for the whole protein chain based on its amino acid sequence. This method correctly predicted the domain boundaries for about 60 % of the proteins analyzed [2].

A method known as DomCut [3] has been developed to predict linker regions among domains based on differences in amino acid composition between the domain and linker regions. The sensitivity and the selectivity achieved using this method were 53.5 % and 50.1 %, respectively. CHOPnet, by Liu et al. [4], uses evolutionary information, predicted secondary structure, solvent accessibility, amino acid flexibility, and amino acid composition to predict domains in protein chains by removing noisy peaks from the raw outputs of neural network classifiers via postprocessing methods. A prediction accuracy of 69 % among all proteins investigated was reported for this approach.

The Armadillo domain predictor software [5] uses an amino acid index to convert a protein sequence to a smoothed numeric profile from which domains and domain boundaries are predicted. The amino acid index derived in that work was named the domain linker propensity index (DLI), and was derived from the amino acid compositions of domain linkers using a nonredundant structure dataset. The software was reported to achieve 37 % sensitivity for multidomain proteins.

The position-specific scoring matrix (PSSM) of the target protein, which can be obtained via PSI-BLAST [6], has been used for domain boundary prediction by PPRODO [7], in combination with an artificial neural network classifier. The overall accuracy of domain boundary prediction as achieved using PPRODO was 67 %.

Among the various protein domain prediction methods developed recently, DOMpro [8] is one of the most important. It employs machine learning algorithms in the form of recursive neural networks to predict domains in a protein chain. Moreover, it utilizes evolutionary information (in the form of profiles), predicted secondary structures, and predicted solvent accessibility of the protein chains. A curated dataset derived from the CATH database was used to test the prediction accuracy of DOMpro. A domain prediction accuracy of 69 % for a combined dataset of single- and multidomain proteins was reported for DOMpro.

In the work of Sikder and Zomaya [9], the performance of DomainDiscovery with respect to assigning protein domain boundaries was significantly improved by including interdomain linker index values, the PSSM, predicted secondary structures, and solvent accessibility information. A support vector machine (SVM) classifier was used to predict the domain boundaries of target sequences. A unique dataset was built for this purpose, based on the principle of consensus among experts regarding domain definition for protein structures. The method was reported to achieve 70 % accuracy for multidomain proteins. A protein domain prediction approach (SSEP-Domain) founded on secondary structure element alignment (SSEA) and profile–profile alignment (PPA) has been proposed by Gewehr and Zimmer [10]. SSEA is useful for rapidly screening potential domain regions, while PPA provides the necessary specificity to select significant hits. A preliminary version of SSEP-Domain was ranked among the top-performing domain prediction severs in the CASP-6 and CAFASP4 experiments.

Cheng [11] proposed a hybrid domain-prediction web service called DOMAC which integrates template-based and ab initio methods. DOMAC predicts the domains in proteins with homologous template structures found in the Protein Data Bank [12]. If a significant homologous template is not found, DOMAC utilizes the ab initio domain predictor DOMpro to predict domains. The preliminary version of the DOMAC server was ranked among the top domain prediction servers in the CASP7 experiment in 2006. However, its performance was very likely overestimated [11]. Since then, DOMAC has also been evaluated on a larger, more balanced, higher quality dataset that was manually curated by Holland et al. [13]. As a result, the overall domain number prediction accuracies achieved using the template-based and ab initio methods were found to be 75 % and 46 %, respectively.

To achieve more accurate and stable predictive performance, a new machine-learning-based domain predictor, DomNet [14], was trained using a novel compact domain profile, predicted secondary structure, solvent accessibility information, and the interdomain linker index. The accuracy of DomNet in benchmark test datasets was observed to be 71 %. FIEFDom [15] is other type of multidomain prediction tool where predictions are obtained using a fuzzy mean operator. This fuzzy operator assigns a membership value to each residue relating to whether it belongs to a domain boundary, and can therefore find contiguous boundary regions. Eickholt et al. proposed a new method, DoBo [16], which uses a machine learning approach with evolutionary signals. It first extracts putative domain boundary signals from MSA between a sequence and its homologs. Those sites are then classified by SVM, with sequence profiles, secondary structures, and solvent accessibility used as features. A recall of 60 % and a precision of 60 % precision were obtained upon applying DoBo to test datasets.

Another SVM predictor, DROP [17], which was trained with 25 optimal features, distinguished linkers from nonlinkers effectively using a two-step feature-selection procedure. In the first step, a random forest algorithm was used to evaluate 3000 features. In the next step, a selection protocol was used to choose optimal features. Applying a creative hinge region strategy, DomHR [18] can predict domain boundaries by constructing profiles of domain hinge-boundary features.

Many contact prediction tools also provide good domain boundary prediction results. In the work of Sadowski [19], kernel smoothening and methods based on building alpha-carbon models were used to obtain contact information. A template-based method, ThreaDom [20], recently proposed by Xue et al., extracts protein domain-boundary information from multiple threading alignments. This method uses domain conservation scores to combine information from template domain structures and terminal and internal alignment gaps.

Overall, existing methods of protein domain prediction can be summarized as follows. First, the prediction of domain boundaries is viewed as a binary classification problem for each residue along a one-dimensional protein chain. Each residue is considered to belong to a domain boundary or not [8]. However, true domain-boundary definitions have not been used in a stricter sense. For example, some domain-boundary prediction works [4, 8] have considered residues within ±20 amino acids of the true domain boundary. Galzitskaya et al. [2] assumed that boundary prediction was successful when the predicted domain boundary fell within ±40 residues of the true domain boundary assigned by SCOP. Armadillo [5] performs residue-based prediction by deriving a residue-based linker propensity index, But its prediction conventions had been relaxed with the assumption that the predicted domain linker were found to be correct when the predicted domain linker overlapped wholly or in part between the correct linker boundaries plus a ±20 residue margin of error added to each boundary.

Secondly, the evaluation and comparison of domain predictors are complicated by the existence of several domain datasets [4] and their domain/linker definitions. Thus, the performance of a predictor when applied to a particular dataset is considerably influenced by the percentage of agreement between the training and test datasets. Finally, some predictors use the specificity and sensitivity of boundary residues [8, 5, 16] whereas others use measures such as the precision of boundary placement or PBP [9] or the normalized domain overlap or NDO [17] for performance evaluation.

In this paper, we present a residue-level prediction of domain/linker annotations for domain-boundary prediction in protein chains. The method that we have developed avoids artificial expansion of the boundary residues, as employed in earlier works, and uses true domain annotations from benchmark databases. The first objective of the work presented in this paper was to assess the strength of the designed feature set using six different machine-learning classifiers for domain residue prediction by performing detailed cross-validation experiments with the benchmark CATH database. Another important objective was to implement the *n*-star quality consensus approach for combining and improving the performances of single-best classifiers in domain-boundary prediction for independent CASP targets. In the following, we first describe the design of the feature set, classifiers, and the consensus approach used in our method. We then review the experimental results obtained using it, discuss those results, before finally drawing some conclusions.

## Materials and methods

In this work, we examined six different machine-learning algorithms using a carefully chosen feature set consisting of a hydrophobicity index, a linker index, polarity values, ordered/disordered regions in the protein sequence, and flexibility parameters for residue-level protein domain boundary prediction from sequence information. We extracted overlapping sequence motifs from the primary sequences and extracted features for all the residues in order to facilitate the selection of the central residue of the sequence fragment. This method is popularly called the sliding window technique, where subsequences are extracted along the complete protein chain, with each residue being positioned at the center once. However, given the width of the sliding window, some of the terminal residues are excluded from the decision process. One of the major issues with this type of sliding window technique is the appropriate selection of the window width. To choose an optimal window of fixed width, it is necessary to experiment on the size of the window which gives best prediction results on linkers and domains. In order to get optimal window we varied the window size at initial part of the experiment. This is often an interesting indicator that shows the extent of the effect the neighboring residues have on the central residue of the subsequence.

A number of existing methods use sliding windows of different widths for domain-boundary prediction. For example, Galzitskaya et al. [2] used a window size of 27 for multidomain proteins. Domcut [3] used a 15-residue window, and PPRODO [7] varied its window size from 17 to 33; optimal performance was reported to occur when using the 25-residue window. These studies motivated us to test the predictive performance of our PDP-CON method with residue windows of various sizes. Window size was varied from 13 to 29, with the optimum predictive accuracy achieved using a 17-residue window (see the “Results” section for details).

Carefully chosen features are extracted for each residue within the selected residue window and then fed as input to a classifier. The pattern classifier then decides on the class of the central residue (i.e., whether it is part of a domain or not—whether it is a domain or linker residue). This process is repeated for the whole protein chain of interest, and the domain regions (and the linker regions—i.e., the boundaries) in the chain are identified. In the following, we first describe the feature set used under this study and then briefly discuss the classifiers considered in order to evaluate the performance of the PDP-CON method. We also discuss the design of the quality consensus strategy, which combines the final decisions of the various classifiers.

### The feature set

We explored various feature sets from the existing literature on domain boundary prediction, and five types of features—predicted ordered or disordered region, normalized flexibility parameter (B-value), polarity values, linker index, and the modified Kyte–Doolittle hydrophobicity scale—were finally chosen for this work. Four features were extracted by exhaustively searching through the 544 features available in the AAindex database, release 9.0 [21] (http://www.genome.jp/aaindex/) (see Table 1). All of the features were evaluated for each residue in the 17-residue window. The rationale behind the selection of the four feature descriptors shown in the table is discussed in subsequent sections.

**Table 1:** Brief description of the features selected from the AAindex database

AAindex accession number	Brief description of feature
VINM940101	Normalized flexibility parameter (B-value), average [22]
GRAR740102	Polarity [23]
JURD980101	Modified Kyte–Doolittle hydrophobicity scale [24]
BAEK050101	Linker index [25]

#### Predicted ordered or disordered region

The distribution of predicted disordered and ordered regions in a multidomain protein has been found to be a good approximation of the arrangement of domains in the three-dimensional structure of the protein. From experimental findings, it is known that large ordered regions separated by smaller disordered regions are likely to be separate domains [26], while the disordered local sequence segments are likely to be linker regions or inter domain spacers between the protein domains. In the present work, the Disprot tool [26] was used to predict the ordered and disordered regions in the protein chains, and these predicted results were used as a feature.

#### Normalized flexibility parameter (B-value)

The presence of multiple domains in proteins gives rise to a great deal of flexibility and mobility, and thus to protein domain dynamics [27]. Domain motions can be inferred by investigating how the structure of the protein of interest varies depending on its environment. The structural flexibility of proteins facilitates various biological processes such as molecular recognition and catalytic activity. Flexible regions are considered to be natively unfolded. The Debye–Waller factor (B-value), which measures local residue flexibility, is widely used to measure residue flexibility. Predicting flexibility may help to unravel protein function. In this work, we used values of the normalized average flexibility parameter (B-value) from the AAindex dataset (accession number: VINM940101) as a features, as the presence of multiple domains increases protein flexibility.

#### Polarity

The distribution of polar and nonpolar side chains is one of the most important factors governing why a protein folds into a particular 3D structure [28]. As the domains are the units of this 3D structure, polarity was another feature from the AAindex dataset (accession number: GRAR740102) to be used in this work.

#### Amino acid linker index

A parameter called the linker index was devised by Sumaya and Ohara to account for the preference for amino acid residues in linker regions [3]. The linker index *S*_i_ for amino acid residue *i* is defined as follows:

$$ {S}_i=- \ln \frac{f_i^{\mathrm{linker}}}{f_i^{\mathrm{domain}}}, $$

where *f*_*i*_^linker^ and *f*_*i*_^domain^ are the frequencies of occurrence of amino acid residue *i* in the linker and domain regions, respectively. A negative value of *S*_*i*_ implies that amino acid residue *i* is more likely to be found in a linker region. In order to include this information in our experiment, the linker index from the AAindex dataset (accession number: BAEK050101) was used as a feature in our work.

#### Hydrophobicity

Studies suggest that the unique 3D structure of a protein is a result of a balance between the stabilizing effect of attractive native interactions and the loss of conformational entropy upon forming those interactions (i.e., upon generating the 3D structure). More specifically, the topology of the chain determines how much entropy is lost when the native interactions are formed. A protein region that has higher entropy when unfolded will form many residue–residue interactions to compensate for the loss of entropy that occurs during folding. On folding, each protein region represents a well-structured part of the globule (i.e., a domain unit). Much of the loss of conformational entropy that occurs upon folding is due to the restrictions on the movement of side chains in the folded protein. Folding occurs because there is a drive to reconfigure the protein such that the hydrophobic side chains are buried inside the molecule so that they avoid contact with the aqueous environment of the protein. The average hydrophobicity for linkers has been found to be 0.65 ± 0.09. Small linkers show an average hydrophobicity of 0.69 ± 0.11, while large linkers are more hydrophobic: 0.62 ± 0.08 [29]. The more exposed the linker, the more likely it is to contain hydrophilic residues. Increasing the number of linker connections between two domains increases the hydrophobicity. To utilize this characteristic, the modified Kyte–Doolittle hydrophobicity scale from the AAindex dataset (accession number: JURD980101) was used as a feature in the current work.

### Design of the classifiers

In this work, we considered six different types of classifiers: decision tree (DT), Gaussian naïve Bayes (GNB), linear discriminant analysis (LDA), support vector machine (SVM), random forest (RF), and multilayer perceptron (MLP). Each type of classifier was trained using threefold cross-validated training data from the CATH database (see the “Results” section for detailed discussion of the training database), resulting in three classifiers of each type (one for each cross-validation experiment), meaning that 18 (= 6 × 3) classifiers were obtained overall. In the next step, an *n*-star consensus strategy (*n* = 3 in this work because the number of classifiers of a particular type is 3) was applied [30] to the three classifiers of each type. Thus, we obtained 1-star, 2-star, and 3-star classifiers of each type.

#### The classifier based on the decision tree technique

The decision tree [31] is a supervised-learning-based classification technique. In it, all of the features are assumed to have finite and discrete domains. A single target feature is termed the *classification*, and each element of the domain of the classification is referred to as a *class*. A decision tree is also called a classification tree; in it, there is a root node that has no incoming edges, and each internal (nonleaf) node represents an input feature. In a decision tree, each internal node splits the instance space into two or more subspaces according to a certain discrete function of the input attributes. In this work, the instance space is partitioned according to the value of a single attribute. For the numeric attribute having a set of possible values (e.g. hydrphobicity, polarity), splitting condition is considered to be a possible value. The arcs from a node corresponding to a feature are labeled with each possible value of the feature. Each leaf of the tree is labeled with a class or a probability distribution of classes.

#### The Gaussian naïve Bayes classifier

In machine learning, naïve Bayes classifiers are a family of simple probabilistic classifiers based on Bayes’ theorem, which assumes that the value of a particular feature shows strong (naïve) independence in the presence or absence of any other feature, given the class variable. As independent variables are assumed, only the variance is determined for the variables of each class. As a result, a small amount of training data is sufficient to be able to estimate model parameters; for example, class priors and feature probability distributions can be estimated with relative frequencies from the training set. These are maximum likelihood estimates of the probabilities. If the probability of each class is assumed to be equal, then a class’s prior may be calculated from probabilities (i.e., priors = 1 / (number of classes)). The class probability can also be estimated from the training set (i.e., (prior for a given class) = (number of samples in the class) /(total number of samples)). To estimate the parameters for a feature’s distribution, one must assume a distribution or generate nonparametric models for the features from the training set [32]. The assumed feature distributions are called the event model of the naïve Bayes classifier. If the features are discrete in form, multinomial and Bernoulli distributions are popular. When dealing with continuous data, it is assumed that the continuous values for each class are distributed according to a Gaussian distribution. For any continuous attribute *x*, then it is necessary to compute the mean and variance of *x* in each class. Let *µ*_*c*_ be the mean of the values of *x* associated with class *c*, and let *σ*_*c*_^2^ be the variance of the values of *x* associated with class *c*. Then, the probability density of some value of *x* associated with class *c*, *P*(*x* = *v*|*c*), can be computed by inserting the value of *v* into the equation for a normal distribution parameterized by *µ*_*c*_ and *σ*_*c*_^2^. That is, $p\left(x=v\Big|c\right)=\frac{1}{\sqrt{2\pi {\sigma}_c^2}}{\mathrm{e}}^{-\frac{{\left(v-{\mu}_c\right)}^2}{2{\sigma}_c^2}}.$

#### The classifier based on linear discriminant analysis

Linear discriminant analysis [33] is a classifier that can be used to find a linear combination of features for separating two or more classes of objects or events. LDA can be applied when the measurements obtained for independent variables during each observation are continuous quantities. The equivalent technique for categorical independent variables is discriminant correspondence analysis. A set of features $\overrightarrow{\boldsymbol{x}}$ for each sample of an object or event of known class *y* is called the training set. The classification problem is then to find a good predictor of class *y* of any sample from the same distribution (not necessarily from the training set), given only an observation $\overrightarrow{\boldsymbol{x}}$ [34]. LDA assumes that the conditional probability density functions ($\boldsymbol{p}\left(\overrightarrow{\boldsymbol{x}}\Big|\boldsymbol{y}=0\right)$ and $\boldsymbol{p}\left(\overrightarrow{\boldsymbol{x}}\Big|\boldsymbol{y}=1\right)$ are both normally distributed with mean and covariance parameters of $\left({\overrightarrow{\boldsymbol{\mu}}}_0,{\boldsymbol{\varSigma}}_0\right)$ and $\left({\overrightarrow{\boldsymbol{\mu}}}_1,{\boldsymbol{\varSigma}}_1\right)$, respectively. Under this assumption, the Bayes optimal solution is to predict that points are from the class (y=1) if the log of the likelihood ratios is below some threshold *T*, such that ${\left(\overrightarrow{\boldsymbol{x}}-{\overrightarrow{\boldsymbol{\mu}}}_0\right)}^{\boldsymbol{T}}{\boldsymbol{\varSigma}}_0^{-1}\left(\overrightarrow{\boldsymbol{x}}-{\overrightarrow{\boldsymbol{\mu}}}_0\right)+ \ln \left|{\boldsymbol{\varSigma}}_0\right|-{\left(\overrightarrow{\boldsymbol{x}}-{\overrightarrow{\boldsymbol{\mu}}}_1\right)}^{\boldsymbol{T}}{\boldsymbol{\varSigma}}_1^{-1}\left(\overrightarrow{\boldsymbol{x}}-{\overrightarrow{\boldsymbol{\mu}}}_1\right)- \ln \left|{\boldsymbol{\varSigma}}_1\right|<T$. If no further assumptions are made, the resulting classifier is referred to as quadratic discriminant analysis.

#### The support vector machine classifier

Support vector machines [35] are well-known tools used for two-class pattern classification and linear regression. An SVM will attempt to construct a hyperplane representing a decision surface where the margin of separation between the positive and negative instances is maximized. SVMs rely on preprocessing the input vector and mapping the input pattern to a higher-dimensional space. Using an appropriate nonlinear mapping function *ϕ*(.), it becomes possible to find a hyperplane separating the two classes which may not be possible when the input vector is in its original lower-dimensional space. The goal of SVM training is to find the hyperplane with the largest margin of separation, i.e., the distance between the hyperplane and the nearest training patterns (also called the support vectors) in the higher-dimensional space. Thus, the support vectors are the training patterns that define the hyperplane, and they are the most informative patterns.

Suppose that a training data set *T*_D_ consists of pairs {(***x***_***i***_, ***y***_***i***_), ***i*** = 1, 2 … ***n***, ***x***_***i***_ ∈ ***R***^***n***^  and ***y***_***i***_ ∈ {−1, 1}, where *x*_*i*_ denotes the input feature vector for the *i *th sample and *y*_*i*_ denotes the corresponding target value. For a given input pattern*x*, the decision function of an SVM binary classifier is defined as

$$ f(x)= sign\left({\displaystyle {\sum}_{i=1}^n}{y}_i{\alpha}_iK\left(x,{x}_i\right)+b\right),\mathrm{where}\; sign(u)=\left\{\begin{array}{cc}\hfill 1\hfill & \hfill \mathrm{f}\mathrm{o}\mathrm{r}\;u>0\hfill \\ {}\hfill -1\hfill & \hfill \mathrm{f}\mathrm{o}\mathrm{r}\;u<0\hfill \end{array}\right. $$

where *b *is the bias, *α*_i_ is the Langrange multiplier, and *K*(*x*, *x*_*i*_) is the kernel function. The kernel function is used to map the input feature vector *x* into higher-dimensional feature space to make them linearly separable. Several numbers of kernels are used in support vector machine models. Some of the more popularly used kernel functions are shown below:

- *Gaussian (radial basis function) kernel*: *K*(*x*, *x*_*i*_) = exp(−*γ* * ||*x* − *x*_*i*_||^2^) where $\boldsymbol{\gamma} =\frac{1}{2{\boldsymbol{\sigma}}^2}$ and *σ* is the standard deviation of the *x*_*i*_ values.
- *Polynomial kernel*: *K*(*x*, *x*_*i*_) = (*x*^*T*^*x*_*i*_ + 1)^*d*^, where *d* is the degree of the polynomial.
- *Linear kernel: K*(*x*, *x*_*i*_) = *x*^*T*^*x*_*i*_.

#### The classifier based on a random forest

Random forests [36] are one type of ensemble learning method used for classification (and regression). They combine the decisions of multiple decision trees at training time, where the decisions of all decision trees in the ensemble are apportioned equal weight. As a result, this algorithm combines random decision trees with *bagging* to achieve very high classification accuracy:

- *Input*: a set of *d* training tuples; *k*, the number of models in the ensemble decision-tree algorithm
- *Output*: a composite model, M*
- For each model, create bootstrap sample *D*_*i*_ by sampling *D* with replacement, and then use *D*_*i*_ to derive a model *M*_*i*_
- Let each of the *k* models predict a value for *X* and return the average predicted value.

#### The multilayer perceptron

The multilayer perceptron (MLP) [37] is a special kind of feed-forward artificial neural network (ANN). ANNs model the learning and generalization abilities of the biological neural networks present in human brains. In an MLP, the functions of a biological nerve cell or neuron are modeled by an artificial neuron. To model the triggering action of a biological neuron, an artificial neuron computes a sigmoid function that produces a high value when the sum of its weighted inputs exceeds some threshold. An artificial neuron may have a number of inputs and a single output.

In an MLP, the artificial neurons are arranged in layers such that all the outputs of one layer are connected (through weighted links) to the inputs of each neuron in the next layer. The neurons that receive the inputs supplied to the MLP form the input layer, whereas the neurons that produce the outputs of the MLP form the output layer. The other layers of the MLP—the layers between the input and output layers—are called hidden layers. When an MLP is used as a classifier, as in the present case, its input layer contains the same number of neurons as there are features (representing each piece of input data), i.e., a window of residues in the present case. The output layer of the MLP contains as many neurons as the number of data classes to be handled. The number of hidden layers, the number of neurons in each hidden layer, and the values of the weights associated with the links in the MLP are determined at the time of training. Training is required for an MLP to tune its weight values and the other parameters (including the number of hidden layers and the number of neurons in each hidden layer; see Table S1.2 in the “Electronic supplementary material,” ESM) such that it will respond appropriately to a fixed set of labeled data called the training set. For the work reported here, a single-layer MLP (i.e., an MLP with one hidden layer) was used. According to the universal approximation theorem [38], a single hidden layer is sufficient to compute a uniform approximation to a given training set. A back-propagation learning [39] algorithm is applied to train an MLP. Once the MLP has been successfully trained, it is expected to respond appropriately to a separate dataset termed the test set. This is possible because of the ability of the MLP to generalize. The test data should also be labeled so that the responses of the MLP to it can be judged.

#### The *n*-star quality consensus approach

Here we define a *n*-star quality consensus scheme as *C*_*n*_^*N*^, where *N* is the number of classifiers of a particular type that participate in the specific consensus strategy and *n* (1 ≤  *n* ≤  *N*) is the quality of prediction [30]. Thus, in a 1-star prediction, one of the *N* possible classifiers has predicted the test residue is positive (i.e., it that it is a domain region). Therefore, in an *n*-star consensus scheme, increasing the value of *n* increases the number of classifiers that must predict a positive result before the overall result is considered to be positive. Following this principle, we applied a 3-star quality consensus (*C*_*n*_^3^) approach over three variations of training on threefold cross-validation data for each type of classifier.

## Results

The current experiment aiming at the consensus prediction of domain/linker residues in protein sequences was conducted in two stages. In the first stage, 354 protein sequences in the CATH database (version 2.5.1) were used to perform a threefold cross-validation experiment. In each experimental “fold,” 67 % of the positive/negative samples were used for training and the rest of the samples were used for testing. All six types of classifier (DT, GNB, LDA, SVM, RF, and MLP) were trained in a similar way in order to generate three trained classifiers of each type from the three cross-validated experiments. As a result, 18 (= 6 × 3) classifiers were obtained in total for subsequent use in the consensus approach.

In the second stage of the experiment, we applied a consensus approach to the outputs of the trained networks to generate test results for 109 protein sequences from the CASP-8 dataset [40], 100 protein sequences from the CASP-9 dataset [41], and 59 protein sequences from the CASP-10 dataset [42]. For each type of classifier, 1-star, 2-star, and 3-star consensus classifiers were designed. The following subsections discuss the evaluation metrics, the detailed experimental protocol, and the results obtained from these two experimental stages.

### Evaluation metrics

The design of the appropriate performance evaluation metrics is a key issue in any computational approach. As discussed in the “Introduction,” evaluating and comparing domain predictors is a complex and difficult task because of significant differences between domain datasets and their domain/linker definitions. Some predictors [8, 5, 16] use specificity and sensitivity as standard metrics, whereas others use the precision of boundary placement (PBP) [9] or the normalized domain overlap (NDO) [17] for performance evaluation. However, irrespective of the methodology, the first issue is to define positive and negative samples. Since our work took a residue-level prediction approach, the ground truths were generated accordingly. For example, if we consider a multidomain protein sequence that is annotated as {LLLDDDDDDDDLLDDDDDLL}, where D represents a domain residue and L represents a linker or non-domain residue, then we considered the domain residues to be “positive” and the rest to be “negative” samples during the experimentation. Therefore, in this sliding-window-based prediction scheme, we attempted to predict the annotation of the central residue of a sequence fragment based on the feature vector computed over all of the residues in the fragment. Now, in a practical situation, the number of domain residues is much higher than the number of linker residues. In fact, in many protein sequences, all the residues are annotated as domain residues. This makes it difficult to assess system performance, especially in comparison with other methods.

In spite of the aforementioned problems, standard evaluation measures were employed to check the performance of the present technique. The formulae for the accuracy, recall, precision, and *F*-measure are shown below:

$$ \begin{array}{lll}\mathrm{Accuracy}\ (A)=\frac{\mathrm{TP}+\mathrm{T}\mathrm{N}}{\mathrm{TP}+\mathrm{T}\mathrm{N}+\mathrm{F}\mathrm{P}+\mathrm{F}\mathrm{N}}\hfill &; \hfill & \mathrm{Recall}\ (R)=\frac{\mathrm{TP}}{\mathrm{TP}+\mathrm{F}\mathrm{N}}\hfill \\ {}\mathrm{P}\mathrm{recision}\ (P)=\frac{\mathrm{TP}}{\mathrm{TP}+\mathrm{F}\mathrm{P}}\hfill &; \hfill & F-\mathrm{measure}=\frac{2\times \mathrm{recall}\times \mathrm{precision}}{\mathrm{recall}+\mathrm{precision}}\hfill \end{array} $$

where TP is the number of true positives, FP is the number of false positives, TN is the number of true negatives, and FN is the number of false negatives. The recall (*R*) corresponds to the percentage of the predictions that are correct, precision (*P*) measures the percentage of predicted positives that are truely positive out of predicted positive data. The recall measures how accurately the classifier can classify positive data out of all positive data. Precision measures how precise the data classified as positive by the classifier i.e., it measures the proportion of actual positive data among the predicted positive data. However, considering that the number of positive residues is much larger than the number of negative residues, if FN tends to zero, the recall may tend to 1. Likewise, in the case of sequences in which all of the residues are annotated as domain residues, FP tends to zero and precision may tend to 1. Both of these situations are undesirable because they may not reflect the true strength (or weakness) of the developed prediction system. Accuracy is a better estimate than recall or precision alone, as it reflects the prediction strength in terms of TP and TN combined.

### Three-fold cross-validation experiment with the CATH database

The performance of the developed protein domain/linker residue prediction technique was first validated using the curated CATH database, version 2.5.1. The pairwise identity of proteins in the CATH database is less than 25 %. This database has been used for performance evaluations of many of the recently developed domain prediction techniques, such as DOMPro, Armadillo, and CHOPNet. When the sample was positive, the central residue of the window belonged to a domain region. The other samples were considered to be negative. The complete dataset was divided into three mutually exclusive subsets. In any given cross-validation experiment, two subsets were combined to form the training dataset, while the other subset was considered the test dataset. In this way, three cross-validation experiments were performed using 354 proteins in the CATH database.

#### Determining the optimal window size

In sequence-based prediction, the length of the sequence fragment whose central amino acid is classified as either a domain or a linker residue is crucial. To determine the optimal window length, we chose one standard classifier (MLP) from among the available six different classifiers utilized in this work, and used that classifier to perform exhaustive experiments with various window sizes. For window sizes of 13, 15, 17, 19, 21, 25, and 29 residues, the area under the ROC (AUC) scores were calculated (see Table S1.1 in the ESM). Experimental observations showed that the predictive performance of the classifier was highest for a window size of 17. Therefore, a 17-residue-long window was chosen for use in all of the other experiments presented in this paper.

#### Performance evaluations of the six different classifiers

As discussed before, we conducted three-fold cross-validation experiments using the curated CATH database for all six classifiers. For each experimental “fold,” we obtained one trained classifier. Therefore, three classifiers were designed for each classification scheme. For example, three trained classifiers (DT-CSV-1, DT-CSV-2. and DT-CSV-3) were designed using the DT-based classification strategy. Likewise, three trained classifiers are obtained from each of the GNB, LDA, SVM, RF, and MLP classification schemes. Then, to compare the performance levels of the classifiers, the average test performance was obtained for each type of classifier by averaging the performance levels of the three classifiers of each type when they were applied to the independent CASP protein dataset. The classifier performance results are shown in Table 2. It is clear that the classifiers perform well for the CASP-8 and CASP-9 targets, but they performed relatively poorly for the CASP-10 targets. Although there was no overlap between the proteins in the CATH training set and those in the CASP target set, this poor performance for the CASP-10 targets may be due to inadequate training during the cross-validation experiment.

**Table 2:** Average performance of each of the six classifier types for different CASP proteins

Type of classifier	CASP protein dataset	Recall	Precision	Accuracy	*F*-measure
DT	CASP-8	0.7906	0.921	0.7515	0.8473
	CASP-9	0.7735	0.9019	0.7195	0.8181
	CASP-10	0.7667	0.7985	0.69	0.7557
GNB	CASP-8	0.6307	0.939	0.6427	0.7352
	CASP-9	0.5851	0.9097	0.5832	0.6778
	CASP-10	0.614	0.8216	0.6387	0.6712
LDA	CASP-8	0.6446	0.9386	0.6532	0.7453
	CASP-9	0.5957	0.9144	0.5912	0.6859
	CASP-10	0.5839	0.8148	0.6199	0.6516
SVM	CASP-8	1	0.9126	0.9126	0.9506
	CASP-9	0.9998	0.9009	0.9008	0.9392
	CASP-10	1	0.7901	0.7907	0.847
RF	CASP-8	0.9631	0.915	0.9084	0.9476
	CASP-9	0.9842	0.8995	0.8842	0.9269
	CASP-10	0.973	0.7948	0.801	0.8247
MLP	CASP-8	0.3627	0.9095	0.3872	0.5058
	CASP-9	0.386	0.8896	0.4112	0.5167
	CASP-10	0.3876	0.7782	0.4305	0.4805

Upon comparing the performance levels of the different classifiers, we find that the average performance of GNB was lower than that of DT. The predictive power of GNB was higher for CASP-8 than for the other CASP targets. LDA gave results that were more or less comparable with those afforded by GNB, as the recall, precision, accuracy, and *F*-measure values for LDA were close to those of GNB. RF showed an encouraging level of performance when predicting the domain residues in the CASP-8, CASP-9 targets. For CASP-8 and CASP-9 targets its behavior is found to be consistent whereas prediction results are not satisfactory like in other targets. The predictions made by RF and SVM for the CASP targets were generally more accurate than those provided by the other classifiers. From Table 2, it is clear that SVM performed better than RF for most of the targets. However, we must be cautious about these results, as the high bias for positive samples in the training set means that the performance of SVM may suffer due to overfitting, leading to overestimated results. To avoid such overestimated results, we applied a quality consensus approach in this work (as discussed in the section “The *n*-star quality consensus approach”).

**Fig. 1:**
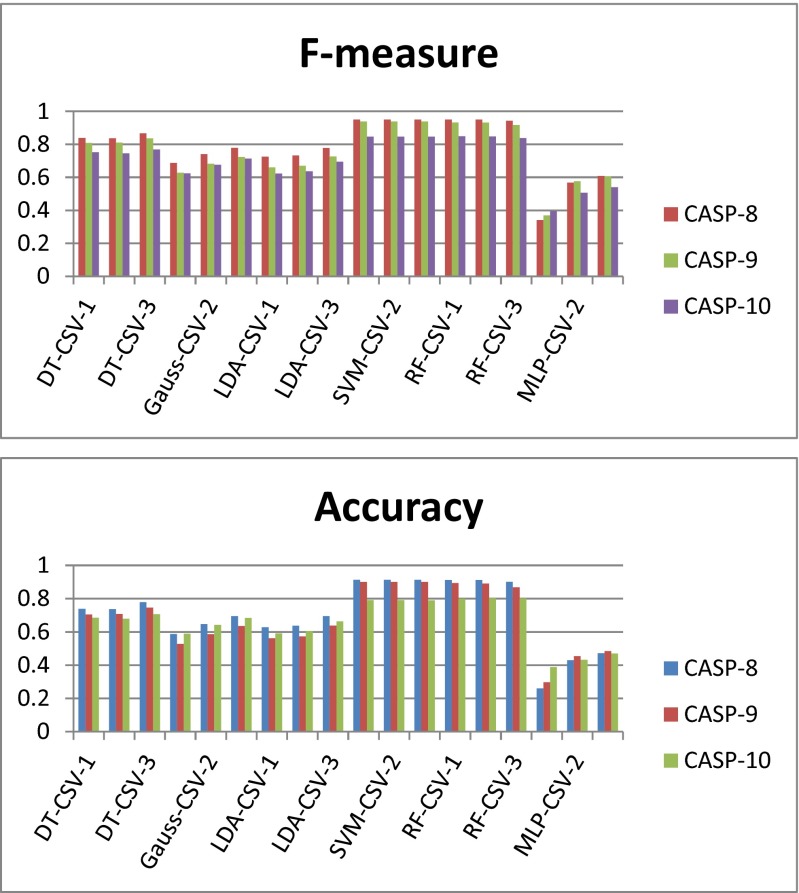
*F*-measures and accuracies achieved by the six types of classifiers on different CASP-8, CASP-9, and CASP-10 protein targets (note that different classifiers of the same type are distinguished by different number suffixes: -1, -2, or -3)

Detailed data on the performance levels of the 18 classifiers generated in this work are given in Tables S5.1–S5.4 in the ESM. The predictions of these classifiers for every protein sequence in each CASP target are shown in Tables S2.1–S2.4, S3.1–S3.4, and S4.1–S4.4 in the ESM. Figure 1 shows the performances of the 18 classifiers (three classifiers of each type) for the CASP targets.

### Consensus predictions for the CASP targets

For each classification scheme, three classifiers were designed based on the cross-validation experiments performed using the CATH database. Utilizing these classifiers, 1-star, 2-star, 3-star consensus strategies were designed according to the definitions given in the “The *n*-star quality consensus approach” section. We should emphasize here that (i) the consensus strategy improves the quality of prediction by combining decisions of multiple classifiers and (ii) applying the consensus approach often improves the performance levels of the individual classifiers. As mentioned previously, a 1-star consensus considers a test sample to be positive if at least one classifier predicts the sample to be positive, but the consensus quality in this case is poor. On the contrary, a 3-star consensus implies that a sample is only considered to be positive if all three classifiers agree on that decision, which corresponds to high consensus quality and consistency among the classifiers in predicting targets. Likewise, a lower 3-star consensus value highlights a lack of consensus among the results predicted by the classifiers. Choosing a 2-star consensus scheme represents a trade-off between the 1-star and 3-star consensus methods.

**Fig. 2:**
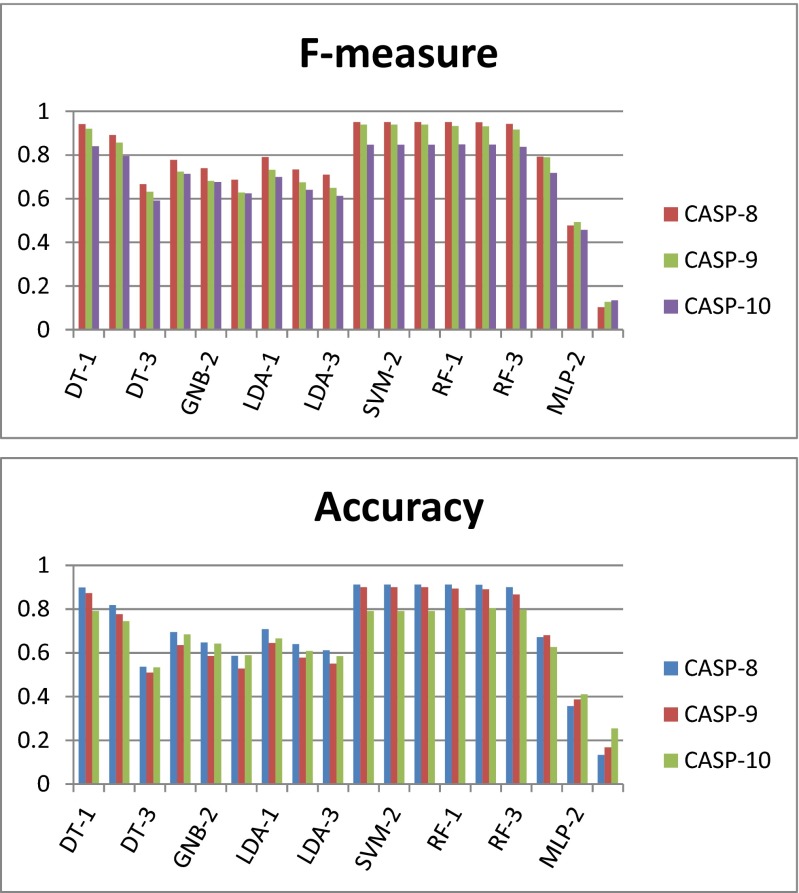
*F*-measure values and accuracies achieved by various consensus classifiers (1-star, 2-star, and 3-star, **as indicated by** the suffixes -1, -2, and -3, respectively, after the abbreviations for classifier types) based on various types of classifier when applied to CASP-8, CASP-9 and CASP-10 protein targets

**Fig. 3:**
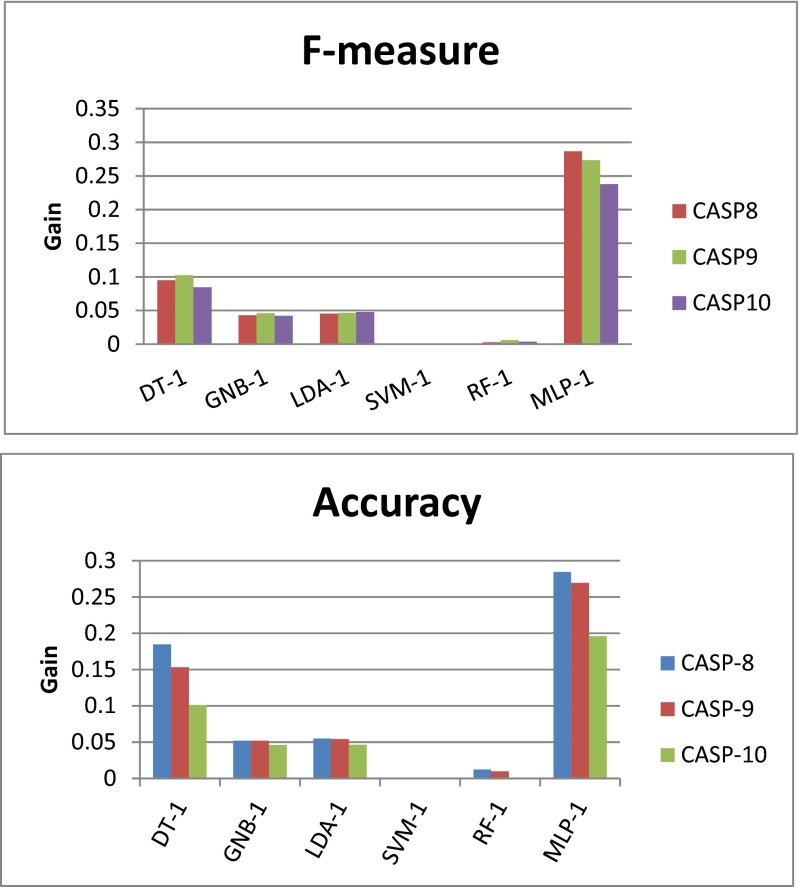
Relative gains in predictive performance (i.e., in *F*-measure values and accuracies) achieved upon applying 1-star consensus classifiers based on various types of classifer to CASP-8, CASP-9 and CASP-10 protein targets, as compared to the corresponding average predictive performance of a single classifier of each type for the same targets

In many cases, we observed improved prediction results upon applying the 1-star consensus scheme than the prediction results provided by the corresponding single classifiers. From Table 3, it is clear that with the introduction of a consensus classifier (1-star), the test performance of each type of classifier (on different CASP targets) is higher than the corresponding results shown in Table 2. When predicting the domain residues in the CASP targets, the *F*-score achieved using DT was 0.09 higher on average upon applying the 1-star consensus scheme. However, using the 1-star consensus scheme did not significantly improve the predictive performance of GNB towards the CASP targets (improvement in *F*-score: 0.04). Consensus LDA classification provides the same performance as GNB. The individual SVM classifiers with an RBF kernel show good predictive power for CASP targets (see the SVM rows in Table 2), and applying the 1-star consensus scheme did not notably improve the predictive power of SVM (see the SVM rows in Table 3). There is a similar situation for RF—the improvements in predictive accuracy achieved upon applying the consensus scheme are marginal. Figure 1 shows the predictive accuracies and *F*-measures of the 18 individual classifiers considered here for the CASP targets. Figure 2 shows the corresponding classifier performance results obtained when 1-star, 2-star, and 3-star consensus schemes were applied to the various classifier types. More detailed results are given in Tables S6.1–S6.4, S7.1–S7.4, S8.1–S8.4 and S9.1–S9.4. For the DT, GNB, LDA, and MLP classifiers, the 2-star and 3-star consensus classifiers show relatively poor performance levels, indicating disagreement between the predictions of the three cross-validation classifiers for each of these classifier types. On the other hand, for SVM and RF, the performance levels of the 1-star, 2-star, and 3-star consensus schemes are very similar, which indicates general agreement between the predictions made by the three classifiers of each type. Figure 3 shows the relative performance gains achieved by applying the different consensus schemes as compared to the performance levels of the respective individual classifiers. The experimental results showed that the individual SVM classifiers already generate very good results, so applying a consensus approach to SVM does not lead to a boost in predictive performance. However, the quality consensus scheme is an important and useful method for predicting test targets. This work presents a range of consensus classifiers (PDP-CON) for residue-level protein domain boundary prediction, and leaves it to the user to choose the one most suited to their specific requirements.

**Table 3:** Average performance levels of the 1-star consensus classifier for each of the six classifier types considered in this work, when applied to predict the domain residues in the proteins in three CASP datasets (see the ESM for a more detailed set of results)

Type of classifier used in 1-star consensus scheme	CASP protein dataset	Recall	Precision	Accuracy	*F*-measure
DT-1	CASP-8	0.9777	0.9157	0.899	0.9423
	CASP-9	0.9701	0.8973	0.8726	0.9206
	CASP-10	0.9698	0.7934	0.7913	0.8405
GNB-1	CASP-8	0.6915	0.9388	0.6947	0.7782
	CASP-9	0.6503	0.9105	0.6353	0.7239
	CASP-10	0.6812	0.8219	0.6847	0.7135
LDA-1	CASP-8	0.7089	0.9386	0.7079	0.7907
	CASP-9	0.6608	0.9144	0.6453	0.7326
	CASP-10	0.6581	0.8155	0.6663	0.6994
SVM-1	CASP-8	1	0.9126	0.9126	0.9506
	CASP-9	0.9999	0.9011	0.9008	0.9393
	CASP-10	1	0.79	0.7907	0.8471
RF-1	CASP-8	0.9987	0.9133	0.9126	0.9504
	CASP-9	0.9979	0.896	0.894	0.9331
	CASP-10	0.9946	0.7928	0.8018	0.8486
MLP-1	CASP-8	0.7074	0.912	0.6717	0.7927
	CASP-9	0.7274	0.8924	0.6808	0.7901
	CASP-10	0.73	0.7834	0.6266	0.7185

### Performance comparison with the state of the art

Before we compare the performance of our domain-residue prediction method with the predictive performance achieved using other methods, we feel that it is important to reiterate that (i) in our residue-level domain/linker prediction approach, we consider all domain residues to be positive and all linkers or non domain residues to be negative, and (ii) it may be unfair to compare different domain-boundary prediction methods based only on recall or precision values. Because they may use different data definitions and evaluation metrics and recall and precision values depend on the number of positive and negative data. As mentioned earlier, in some predictors, the linker annotations have been relaxed, so recall and precision values of these predictors may not present the same meaning for prediction of true domains or linkers. However, we explore this topic in detail below and present a comparative analysis in Table 4. Please note that PDP-CON obtains the true domain-boundary annotations from the CATH database, whereas predictors such as DomPro and PPRODO consider the domain-boundary region to be the residues within ±20 residues of the true boundary. As a result, our training dataset contains unequal proportions of domain and linker residues, and prediction results are often biased towards the majority class (domain residues in this case). We considered the ab initio methods PPRODO [7], DomPro [8], DROP [17], and DoBo [16] in our comparative analysis. PPRODO and DomPRo are not recently developed methods, but they are well-established machine learning based prediction methods, whereas DROP and DoBo are relatively recently devised machine-learning methods that are widely used by the research community.

**Table 4:** Comparative analysis of ab initio methods of protein domain boundary prediction

Predictor name and reference	Training data	Domain boundary annotation	Test data and results
PPRODO [7]	Nonredundant set of 522 proteins	Domain boundary is considered to be ± 20 residues from the true domain boundary; these residues are considered to be positive. The other (nonboundary, i.e., domain) residues are considered to be negative.	CASP-5 10-fold cross-validation experiment Aaccuracy of about 66 %
DOMPro [8]	CATH (2.5.0)		CAFASP-4 Specificity 0.71 Sensitivity 0.71
DROP [17]	DS-All	Linker (nondomain) predictor.	CASP8 NDO score: 0.76
Dobo [16]	CATH(3.3.0)	Domain-boundary definitions from PSI-BLAST	CASP9 Multidomain Recall: 0.72 Precision: 0.68 Accuracy: 0.82
Current work: PDP-CON (1-star consensus SVM)	CATH (2.5.1)	True domain definitions obtained from CATH (2.5.1)	Accuracy: CASP8: 0.91 CASP9: 0.90 CASP10: 0.79 (see Table 3 for details)
Current work: MLP-1 (with *k* = 20)	CATH (2.5.1)	Domain boundary is considered to be ± 20 residues from the domain definition obtained from CATH (2.5.1); domain boundary residues vare considered to be positive and the other (domain) residues are negative	Recall: CASP8: 0.94 CASP9: 0.94 CASP10: 0.96 (see Table 5 for details)

For predictors such as DomPro, PPRODO, and FIEFDom, the domain boundary region is considered to include the residues that are ±20 residues (say, ± *k* residues in general) from the true boundary assignment. This signifies that these methods extend/relax the domain boundary with respect to the true domain boundary. Pre-existing methods differ from our method in two aspects: most of them assume that *k* > 0, i.e., ± *k* residues around the true domain boundary (linker) region are also included in the boundary, and those methods consider residues in the boundary (linker) region to be positive samples, in contrast to our method. However, all pre-existing domain boundary prediction methods (e.g., DomPro, DoBo, PPRODO, DROP, etc.) essentially predict only at the residue level. For example, DomPro is a binary classifier (much like our method) which predicts whether a residue is a boundary residue or not. To make this decision, it considers ± 20 residues around the true boundary residue to be positive samples during training and testing experiments. Some of the techniques also use a post-processing method to identify false-positive residues. Our method, in contrast, uses the actual domain/linker definition (*k* = 0), and the annotated domain residues are considered positive samples. Our method showcases an alternate strategy for preparing training samples for the classifiers and eliminates the need to artificially expand the number of boundary residues (in order to increase the number of positive samples during the training of the classifier). Our strategy, however, has its own limitations, such as the possibility of overfitting the domain residues. To avoid this, different classifiers are repeatedly trained and tested, and the detailed protein-specific prediction results are listed in the ESM.

In an additional performance comparison with our proposed methodology, we also applied a scheme in which *k* = 20 and linker residues were considered to be positive data samples when predicting the boundary regions in benchmark CASP targets and 354 proteins in the CATH database 2.5.1 were employed to train the MLP classifier. The average performance levels of the resulting classifier when it was used on the CASP-8, CASP-9, and CASP-10 target proteins are reported in the ESM as well as Table 5.

**Table 5:** Average performance levels of the MLP-1 classifier with *k* = 20 when applied to three CASP protein datasets (see Table S16a–c in the ESM for protein-specific results)

Target CASP protein dataset	Accuracy	Recall	Precision	*F*-measure
CASP-8	15.71195	0.938722	0.124689	0.220137
CASP-9	26.21427	0.946008	0.247175	0.391943
CASP-10	32.86406	0.957484	0.315238	0.474314

**Fig. 4a–f:**
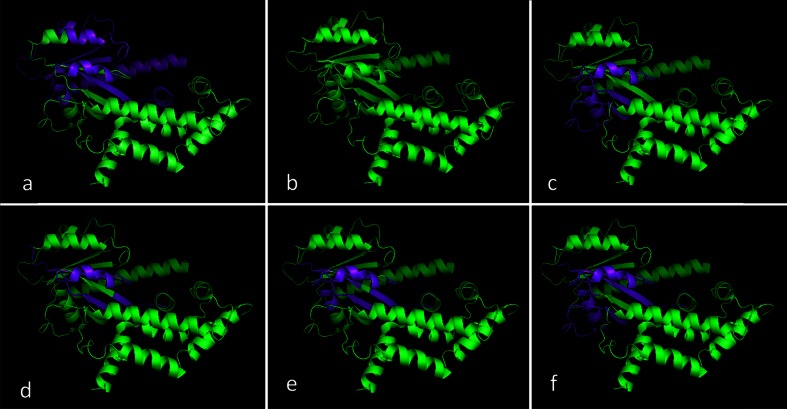
Comparison of various domain/linker prediction results obtained for a sample CASP-8 target protein (T0409): **a** ground truth annotation, **b** results from the DOBO predictor, **c** results from the DROP predictor, **d** results from our LDA-1 predictor, **e** results from the MLP-1 predictor, and **f** results from MLP-1 with *k* = 20. Diagrams were created using the Pymol molecular graphics system; linker residues are shown in *purple* and domain residues in *green*

The performance levels of most of the classifiers when applied to the benchmark CASP targets were noted and compared with those afforded by the PDP-CON classifiers. As reported in the ESM for ThreaDom, the precision values of DROP, DOMPro, and PPRODO upon application to the CASP-9 dataset were found to be 0.679, 0.727, and 0.56, respectively, while the corresponding recall values were 0.26, 0.21, and 0.39. When applied to CASP-10 targets, the corresponding precision values were 0.714, 0.444, and 0.591 and recall values were 0.156, 0.109, and 0.406, respectively. To facilitate a better understanding of the prediction results, a 3-D visualization of the domain/linker annotations (created using PyMol software) obtained by different methods is also included in Fig. 4.

## Conclusion

The work presented in this paper may be considered a quality-consensus based machine-learning method for predicting domain/linker residues in protein chains using a carefully selected set of physicochemical features. The key contributions of our work are as follows: (i) a thorough assessment of different machine-learning classifiers for protein boundary prediction and domain/linker residue prediction, (ii) the selection of the optimal subsequence length for feature extraction, (iii) the identification of effective physicochemical features, and (iv) the utilization of a quality consensus approach that combines the results from different machine-learning methods. We have demonstrated that the developed method should prove useful for the functional annotation of complex protein chains, including protein domain boundary prediction.

Obtaining good performance during binary classification where the proportion of positive and negative samples is significantly skewed is a challenging task. To tackle this problem, six types of classifier were investigated and their power to predict the domain residues in benchmark test proteins associated with the CASP experiments was measured. Moreover, a novel 3-star quality consensus approach was applied to further improve and grade the prediction quality, based on outputs from different variants of the same classification scheme. Experimental observations showed that the designed feature set in combination with the SVM classifier based consensus approach effectively predicted the domain regions in multidomain protein chains. On average, the PDP-CONs were able to predict the domain boundaries in CASP proteins with an accuracy of 88.7 % and an *F*-measure of 92.6 %. The cross-validated experimental setup utilizing the standard CATH database also follows the similar sliding window technique of fixed width and true domain linker annotations. However, we propose that all six of the classification schemes included in our work add great value to the experimental design. Due to skew towards positive samples in the training experiment, the test performance of the SVM classifier can often lead to overestimated results. Therefore, the quality consensus parameters must be tuned (choosing an appropriate value of *n* is often crucial) before acceptable prediction results can be obtained for unknown test samples. Alternatively, we propose that the MLP, RF-, LDA, DT, and GNB-based consensus classifiers should be used in different cases.

In our work, prediction decisions from the three experimental folds were combined to design *n-*star quality consensus strategies. A 3 -star quality consensus scheme was obtained by combining the decisions of the three networks produced in the three sets of cross-validation experiments. In most cases, applying the consensus strategy was found to give better predictive performance than the best individual networks. Using this approach, we can effectively quantify the prediction quality and grade the results based on the choice of *n* in the *n*-star consensus scheme.

One of the initial objectives of our work was to inspect the performance levels of the predictors in the case of true domain definition (i.e., by taking *k* = 0). Another reason behind this choice was our existing expertise in designing effective residue-level features and prediction routines. As a consequence, we marginally shifted from the popular understanding of the boundary/nonboundary prediction problem to a domain/linker prediction problem. This situation led to a data distribution that was skewed in an even more complex manner, and we faced difficulties in handling the unequal proportions of positive (domain or nonboundary residues) and negative (linker or boundary residues) data. Existing predictors also face this problem, but the data distribution improves as *k* is increased. We consider an investigation of the optimum choice of *k* to be one future direction of the research performed here.

Methods for building feature importance rankings based on a random forest can also be used to gain more insight into amino acid properties that are correlated with domain boundaries. To further demonstrate the validity of our method, we also plan to include a comparison of it with other machine-learning algorithms in our next work. In terms of further research, the developed consensus method could be trained on more informed data to improve prediction accuracy and could be used to identify post-translational modification (PTM) sites in proteins based only on local sequence information [43, 44, 30]. In addition, we propose to explore the problem of optimal feature selection for domain boundary prediction by using a feature ranking strategy—possibly a random forest classifier. Finally, please note that the complete database, source code, necessary binaries, and help files relating to this work are available for free download for academic use from our web repository at https://cmaterju.org/cmaterbioinfo/.

## Electronic supplementary material

Below is the link to the electronic supplementary material.

ESM 1 (XLSX 497 kb)
